# Determinants of spring migration departure dates in a New World sparrow: Weather variables reign supreme

**DOI:** 10.1002/ece3.10874

**Published:** 2024-02-22

**Authors:** Allison J. Byrd, Katherine M. Talbott, Tara M. Smiley, Taylor B. Verrett, Michael S. Gross, Michelle L. Hladik, Ellen D. Ketterson, Daniel J. Becker

**Affiliations:** ^1^ Environmental Resilience Institute Indiana University Bloomington Indiana USA; ^2^ Department of Biology Indiana University Bloomington Indiana USA; ^3^ Department of Ecology and Evolution Stony Brook University Stony Brook New York USA; ^4^ School of Biological Sciences University of Oklahoma Norman Oklahoma USA; ^5^ U.S. Geological Survey California Water Science Center Sacramento California USA

**Keywords:** haemosporidians, motus, movement ecology, songbirds, stable isotope

## Abstract

Numerous factors influence the timing of spring migration in birds, yet the relative importance of intrinsic and extrinsic variables on migration initiation remains unclear. To test for interactions among weather, migration distance, parasitism, and physiology in determining spring departure date, we used the Dark‐eyed Junco (*Junco hyemalis*) as a model migratory species known to harbor diverse and common haemosporidian parasites. Prior to spring migration departure from their wintering grounds in Indiana, USA, we quantified the intrinsic variables of fat, body condition (i.e., mass ~ tarsus residuals), physiological stress (i.e., ratio of heterophils to lymphocytes), cellular immunity (i.e., leukocyte composition and total count), migration distance (i.e., distance to the breeding grounds) using stable isotopes of hydrogen from feathers, and haemosporidian parasite intensity. We then attached nanotags to determine the timing of spring migration departure date using the Motus Wildlife Tracking System. We used additive Cox proportional hazard mixed models to test how risk of spring migratory departure was predicted by the combined intrinsic measures, along with meteorological predictors on the evening of departure (i.e., average wind speed and direction, relative humidity, and temperature). Model comparisons found that the best predictor of spring departure date was average nightly wind direction and a principal component combining relative humidity and temperature. Juncos were more likely to depart for spring migration on nights with largely southwestern winds and on warmer and drier evenings (relative to cooler and more humid evenings). Our results indicate that weather conditions at take‐off are more critical to departure decisions than the measured physiological and parasitism variables.

## Graphical abstract

To test for interactions among weather, migration distance, parasitism, and physiology in determining spring departure date, we used Dark‐eyed Juncos (*Junco hyemalis*) as a model migratory species known to harbor diverse and common haemosporidian parasites. The best predictor of spring departure date was average nightly wind direction and a principal component combining relative humidity and temperature. Juncos were more likely to depart for spring migration on nights with largely southwestern winds and on warmer and drier evenings (relative to cooler and more humid evenings).

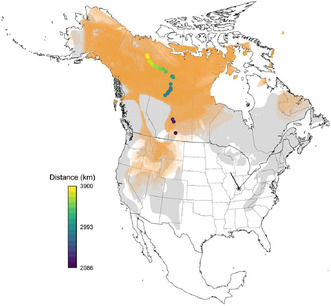

## 1 INTRODUCTION

Over‐wintering duration and timing of departure for avian spring migration is a process influenced by a host of extrinsic and intrinsic factors (Covino et al., 2015; Hurlbert & Liang, 2012; Satterfield et al., 2018). The timing of spring migration must balance the advantages of early arrival on the breeding grounds, including higher quality mates and territories (Gunnarsson et al., 2006; Møller, 1994; Newton, 2010; Rotics et al., 2018; Smith & Moore, 2005), with the risk of arriving before adequate food resources are available (Gunnarsson et al., 2006; Møller, 1994; Newton, 2010; Rotics et al., 2018; Smith & Moore, 2005). Stressors experienced on the wintering grounds can also affect the timing and duration of spring migration. Extrinsic factors such as changing weather conditions (Marra et al., 2005) and intrinsic factors such as high parasite intensity (Dietsch, 2005; Reed et al., 2003) can alter stopover duration as birds recover from or accommodate these additional physiological burdens (Schmaljohann et al., 2009). The relative contribution of extrinsic and intrinsic factors experienced at the wintering grounds in shaping spring departure remains less well‐understood, and studies addressing these interactions are especially valuable as anthropogenic pressures continue to influence the climate and natural habitat (Kubelka et al., 2022; Visser et al., 2009; Wilcove & Wikelski, 2008).

For decades, ornithologists have known that weather variables influence avian migration timing in both spring and fall (see Richardson, 1978). Factors that influence the probability of migratory departure include wind speed (Chapman et al., 2016; Drake et al., 2014; Kemp et al., 2010; Liechti, 2006; Nussbaumer et al., 2022), wind direction (Covino et al., 2015; Hebrard, 1971; Horton et al., 2016; Kemp et al., 2010; Lack, 1963; Lack & Eastwood, 1962; Sinelschikova et al., 2007), temperature (Hüppop & Winkel, 2006; Marra et al., 2005; Saino et al., 2007; Tøttrup et al., 2010; Usui et al., 2017), and relative humidity (Klaassen et al., 2012; Liechti, 2006; Schmaljohann et al., 2009; Serra‐Cobo et al., 1998; Zhang & Wu, 2018). The high correlation among these weather variables adds to the difficulty in disentangling the influence of these factors from potential additional stressors. For example, warm air can hold more moisture than cold air; thus, at the same absolute humidity, cooler air (perhaps counterintuitively) has higher relative humidity than warmer air (Lawrence, 2005).

The relationship between wind and bird migration has been observed for decades, and prevailing winds were a likely a selection factor in that shaping the evolution of migratory patterns (Able, 1973; Alerstam, 1979a, 1979b; Richardson, 1978). Birds that are highly selective of favorable winds can maximize flight speed and minimize energy expenditure across both long‐ and short‐distance migratory flights (Alerstam, 1979a). Migrants have faster ground and air‐speeds in spring migration than fall (Horton et al., 2016), and spring migration is typically completed over a shorter duration than fall (Newton, 2010). As such, models that forecast bird migration rely heavily on wind data (Erni et al., 2002; Van Doren & Horton, 2018).

Intrinsic factors have also been recognized to affect readiness for migratory departure. Given the energetic costs of migration (Wikelski et al., 2003), adequate fat stores are integral in providing fuel for long‐distance flight and larger stores can be a reliable predictor of migratory readiness (Price, 2010; Ramenofsky, 1990; Weber et al., 1994; Witter & Cuthill, 1993); correspondingly, measures of body condition (i.e., mass ~ tarsus residuals) allow for standardized body size comparisons among individuals (Labocha & Hayes, 2012). The degree of physiological stress and immune state of individuals at the wintering grounds can also affect spring migratory timing. Preparation for long‐distance migration increases plasma concentrations of corticosterone (the primary avian glucocorticoid responsible for maintaining homeostasis) in many songbirds (Holberton, 1999; Landys et al., 2004), which can facilitate earlier departure from the wintering grounds or spring stopover (Eikenaar et al., 2013, 2017). Elevated corticosterone can shift the composition of leukocytes in blood, subsequently elevating the ratio of heterophils to lymphocytes (HL ratios; Davis et al., 2008). While plasma corticosterone levels are highly sensitive to the acute stress of capture, stress‐induced changes in leukocyte profiles occur more slowly, such that HL ratios can serve as a more tractable approximation of energetic costs prior to migration (Davis & Maney, 2018). In a similar fashion, the energetic cost of migratory preparation can induce trade‐offs with the immune system (Lochmiller & Deerenberg, 2000), such that migrants may downregulate immune activity or rely primarily on less costly immune defenses. For example, several thrush species show lower total leukocyte counts upon arrival at stopover sites in spring, reflecting such trade‐offs (Owen & Moore, 2008). Variation in immune investment may in turn affect departure decisions; recent work demonstrated that songbirds with higher titers of natural antibodies and immunoglobulin Y have longer spring stopovers (Brust et al., 2022).

Related to seasonal variation in physiological stress and immunity, songbirds may also experience additional overwintering stressors that affect spring migration. For example, exposure to toxins such as neonicotinoids in wintering food sources (Eng et al., 2017) can delay migration departure or increase stopover duration as birds recover from or accommodate these burdens. An additional such stressor could be through parasite infection, such as that from dipteran‐borne haemosporidian blood parasites (i.e., the genera *Plasmodium*, *Haemoproteus*, and *Leucocytozoon*). Experimental infections of captive birds have shown migration‐relevant physiological costs of infection, such as reduced general activity (Mukhin et al., 2016; Yorinks & Atkinson, 2000) and body condition (Atkinson et al., 1995) as well as shifts in the development of migratory restlessness (Kelly et al., 2016, 2020). Observational studies have also identified impacts of haemosporidian infection on body condition in migrating birds (Garvin et al., 2006; Merrill et al., 2018), on the timing of arrival to the breeding grounds (Asghar et al., 2011; Santiago‐Alarcon et al., 2013), and on autumn departure timing (Ágh et al., 2019). However, it remains unclear how haemosporidian infections interact with extrinsic and other intrinsic factors to affect migration timing.

In this study, we used overwintering migratory Dark‐eyed Juncos (*Junco hyemalis*; hereafter “junco”) to test the hypothesis that physiological state and haemosporidian infection interact with weather conditions and migration distance to shape spring migratory departure timing. Haemosporidia have been well‐characterized in Dark‐eyed juncos, with chronic (i.e., long‐term) infections detected in wintering and breeding populations (Becker et al., 2019, 2020; Deviche et al., 2001; Ferrer, 2022; Martínez‐Renau et al., 2022; Slowinski et al., 2018; Talbott et al., 2022). We used stable isotopes of hydrogen from feathers to estimate the distance to breeding grounds for each individual (Bowen et al., 2014; Hobson, 1999; Hobson et al., 2012; Rubenstein & Hobson, 2004; Wunder, 2010) and the Motus Wildlife Tracking System to determine departure date (Taylor et al., 2017). We predicted that birds with higher fat reserves, low intensity or no haemosporidian parasitism, lower HL ratios, and lower total leukocyte counts would depart earlier in the year and during evenings of following winds (i.e., tailwind). We also predicted that wind direction (following winds) would be more important than wind speed and that headwinds would be the strongest deterrent to departure, even more so than intrinsic predictors.

## 2 METHODS

### 2.1 Junco sampling and captive housing

We caught wild juncos from January 31, 2020, to February 26, 2020, at four locations within 15 km of Bloomington, Indiana, USA. None of the birds was captured at the release site, and we systematically rotated capture efforts through all four sites to avoid confounding results by capture date or location. We captured juncos using baited mist nets and walk‐in traps, took standard morphometric measurements including wing, tail, tarsus and bill length, mass, condition, and body fat (Fudickar et al., 2016; Jawor et al., 2006; Singh et al., 2019). We assigned a numerical rank to subcutaneous fat visible beneath the skin as a measure of fat score and assigned a numeric value to flight muscle thickness as a measure of condition (Labocha & Hayes, 2012). We then fit each bird with an aluminum U.S. Fish & Wildlife Service leg band (federal permit # 20261, Indiana state permit 20‐528).

While capture efforts were ongoing, juncos were held at an indoor aviary at Indiana University to prevent additional exposure to arthropod vectors of haemosporidia. Juncos were provided ad libitum water and food (mealworms, organic millet, organic sunflower seeds, and a blended mixture of organic millet, organic carrots, and organic blueberries) and could fly freely in 6.4 × 3.2 m rooms. Light cycles for rooms reflected the natural photoperiod of Bloomington, Indiana, at the time of the study.

As a potential additional extrinsic factor, juncos in this study were randomly dosed with either a neonicotinoid (imidacloprid suspended in sunflower seed oil) or control (sunflower seed oil) to experimentally test impacts on migration timing. However, imidacloprid metabolites of 5‐OH‐imidacloprid and imidacloprid‐olefin were detected in only one bird's post‐dose plasma at 46.67 and 72.23 ng/mL, respectively. This individual was removed from all subsequent analyses. Imidacloprid or metabolites were not detected in pre‐dose or post‐dose (6 h after) plasma of any other individuals (*n* = 37; Table S1). Therefore, we did not include imidacloprid exposure as a possible predictor of migratory timing. Possible explanations for the absence of imidacloprid detection in plasma samples are that the dose was regurgitated or rapidly metabolized by all birds.

On the days of release (March 3, 2020, and March 4, 2020), we attached a nanotag to each bird (Lotek model NTQB2‐2, 11 × 5 × 4 mm, 0.32 g, ≦0.5 g total mass including leg harness). Birds were released at Kent Farm Research Station (located nine miles east of Bloomington, IN), an area frequented by overwintering juncos; we provided organic seed in feeders and on the ground in an attempt to minimize departure from the area due to resource limitation (Bridge et al., 2010).

### 2.2 Hematological analyses

At capture, we collected ≤150 μL blood from each bird by pricking the brachial vein with a sterile needle, followed by collection with heparinized capillary tubes. We also collected the first secondary feather from each bird for stable isotope analysis (Fudickar et al., 2016). We separated plasma and red blood cells (centrifuged for 10 min at 11,180 *g*) and stored samples at −20°C until DNA extraction using a Maxwell RSC Whole Blood DNA Kit (Promega). Any birds unable to be sexed by wing length and plumage were verified using sexing PCR of extracted DNA (Griffiths et al., 1998). Only males (*n* = 37) were included to control for sex differences in migration timing, as female juncos migrate earlier than males (Nolan & Ketterson, 1990).

Because birds were held in captivity for variable lengths between capture and release with nanotags (x̄ = 25, range of 13–33 days), we collected additional blood samples 24 h prior to release, using the blood collection protocol described above. We prepared thin blood smears on glass slides stained with Wright–Giemsa (Quick III, Astral Diagnostics). We then evaluated leucocyte profiles and haemosporidian intensity using light microscopy (AmScope, B120C‐E1). A single observer (TBV) recorded the number of leukocytes under 400× magnification across 10 random fields to estimate inflammatory state and investment in cellular immunity. A differential count was then performed by recording the identity of the first 100 leukocytes (heterophils, lymphocytes, monocytes, eosinophils, and basophils) at 1000× magnification (oil immersion; Campbell, 1995). We then screened 100 fields of view at 1000× magnification for *Plasmodium* spp., *Haemoproteus* spp., and *Leucocytozoon* spp. (Becker et al., 2019, 2020; Cosgrove, 2005; Valkiunas et al., 2008). We derived the mean total leukocyte count, HL ratios, and the total number of haemosporidian‐infected erythrocytes (parasite intensity) as three predictor variables. Usable blood smears were available for 34 of our 37 tagged individuals.

### 2.3 Hydrogen isotope analysis

We used stable isotopic analysis of hydrogen (δ^2^H) in feathers to infer probable breeding latitude and median distance from capture point (Bowen et al., 2014; Hobson, 1999; Hobson et al., 2012; Rubenstein & Hobson, 2004; Wunder, 2010). Feathers were cleaned using a 2:1 chloroform: methanol solution to remove external oils and contaminants. We used a forced isotopic equilibration procedure to ensure exchangeable hydrogen with a water vapor of known isotopic composition in a flow‐through chamber system at 115°C (Sauer et al., 2009; Schimmelmann, 1991). Samples were analyzed using a thermal conversion element analyzer coupled with a ThermoFinnigan Delta Plus XP isotope ratio mass spectrometer at the Indiana University Stable Isotope Research Facility. Isotopic data are reported in standard per mil notation (‰) relative to Vienna Standard Mean Oceanic Water (VSMOW) using two reference materials: USGS77 (polyethylene powder) and hexatriacontane 2 (C_36_ n‐alkane 2). Analytical precision was ±1.0‰ for δ^2^H values. We calculated the isotopic composition of the non‐exchangeable hydrogen per sample, assuming a 17% exchangeability rate for feathers (Schimmelmann, 1991; Schimmelmann et al., 1999).

We performed Bayesian geographic assignments for the breeding location of individual birds based on feather δ^2^H values using the *assignR* package (Chao et al., 2020). To calibrate our precipitation‐feather isoscape, we used growing season precipitation isoscape rasters from waterisotopes.org (Bowen et al., 2005; Bowen & Revenaugh, 2003) and the isotopic composition of non‐migratory Dark‐eyed juncos from previous studies (Becker et al., 2019; Hobson et al., 2012). Median latitudinal and distance estimates were calculated from geographic assignment probability maps by extracting the geographic cell coordinates with the highest posterior probability (top 10%) using the *raster* package (Hijmans, 2019). Migration distance was defined as the distance from the release site to the centroid of the estimated breeding polygon (Becker et al., 2022; Wanamaker et al., 2020).

### 2.4 Motus data processing

Motus data (project 240; available at https://motus.org/data) were downloaded on July 25, 2020, four months after the known period of spring migration in Indiana of wintering juncos (Ketterson & Nolan, 1982). Data were filtered and cleaned following a standard protocol using the *motus* package in R (Taylor et al., 2017). Specifically, we removed runs with a low probability of being a true detection (i.e., motusFilter = 0) and adopted a moderately strict filter to be more conservative about detection inclusions. We then derived the departure date as the last day (ordinal date) a bird was detected at the Kent Farm Research Station Motus station. We additionally report cases in which birds were detected at other Motus stations between banding and our data download date.

### 2.5 Weather data

We obtained relative humidity data from ncei.noaa.gov (Diamond et al., 2013) and all remaining weather variables (humidity, wind speed, wind direction [converted to wind rose] and weather type) from timeanddate.com (Thorsen, 1995–2023). We averaged hourly data from 1800 to 2400 on departure date evenings (thus, variables are labeled average humidity, etc).

Because average relative humidity and average temperature are dependent (Lawrence, 2005), we conducted a principal components analysis (PCA) of these two variables, with variables centered and scaled to have unit variance. The first PC (hereafter weather PC1) explained 56% of the variation and was loaded negatively by average relative humidity (−0.71) and positively by average temperature (0.71); resulting values indicate increasingly warm and dry evening weather. This variable was used in downstream analyses alongside average wind speed and average wind direction.

### 2.6 Statistical analyses

Prior to analyses of migration timing, we used a linear mixed effects model with a random effect of site to derive an index of body condition through the residuals of a regression of mass (measured prior to release) on tarsus length (Schulte‐Hostedde et al., 2005; Wanamaker et al., 2020).

We modeled days until spring migratory departure under an event‐time analysis framework, using additive Cox proportional hazard mixed models (CPHMMs, Therneau & Grambsch, 2000).These semi‐parametric models allow determining how the risk of departure changes with covariates that can also vary with time and thus the terms “risk” or “hazard” are used to accurately describe what is modeled (e.g., “risk of spring migration”). These models have been applied to study migration timing in both avian and non‐avian systems (Castro‐Santos & Haro, 2003; Dossman et al., 2015).We used the *mgcv* package to fit CPHMMs with a random effect of site. To test the direct and interactive effects of extrinsic (i.e., weather PC1, wind direction, wind speed) and intrinsic predictors (i.e., fat, body condition, total leukocytes, HL ratios, haemosporidian intensity) on risk of spring departure (Wood, 2017). Including all predictor variables and biologically relevant interactions in a single full model was not possible given our sample size (*n* = 34, excluding birds without hematology data). We instead built 10 candidate CPHMMs representing a priori hypotheses of additive and interactive effects while restricting models to at most three fixed effects to limit overfitting (Burnham & Anderson, 2002). Most predictors showed low collinearity (*ρ* ranged from −0.63 to 0.62, x̄ = 0.01), and moderately correlated predictors (i.e., weather PC1 and wind speed, *ρ* = −0.63; body fat and condition, *ρ* = 0.62) were excluded from the same models. All predictors were modeled using thin plate splines with smoothing penalty with the exception of wind direction, which used a cyclic cubic spline to account for circular data. Interaction terms were modeled as tensor products. Our models represented additive effects of intrinsic variables only (e.g., fat, haemosporidian intensity, and total leukocytes), additive effects of extrinsic variables only (e.g., wind direction and weather PC1), additive effects of both intrinsic and extrinsic variables (e.g., haemosporidian intensity, HL ratios, and weather PC1), interactive effects of intrinsic variables (e.g., effects of haemosporidian intensity dependent on HL ratios), interactive effects of extrinsic variables (e.g., effects of wind speed depend on wind direction), and interactive effects of intrinsic and extrinsic variables (e.g., effects of weather PC1 depend on haemosporidian intensity).

We compared CPHMMs fit with maximum likelihood using Akaike information criterion adjusted for small sample size (AICc) with the *MuMIn* package (Bartoń, 2013). We also derived Akaike weights (*w*_*i*_) to facilitate comparison and considered models within two ΔAICc of the top model to be competitive. Competitive models were refitted to the full dataset, with CPHMM predictions visualized as relative hazards with 95% confidence intervals (Nakagawa & Schielzeth, 2013).

## 3 RESULTS

### 3.1 Hydrogen isotopes and likely breeding origins

**FIGURE 1:**
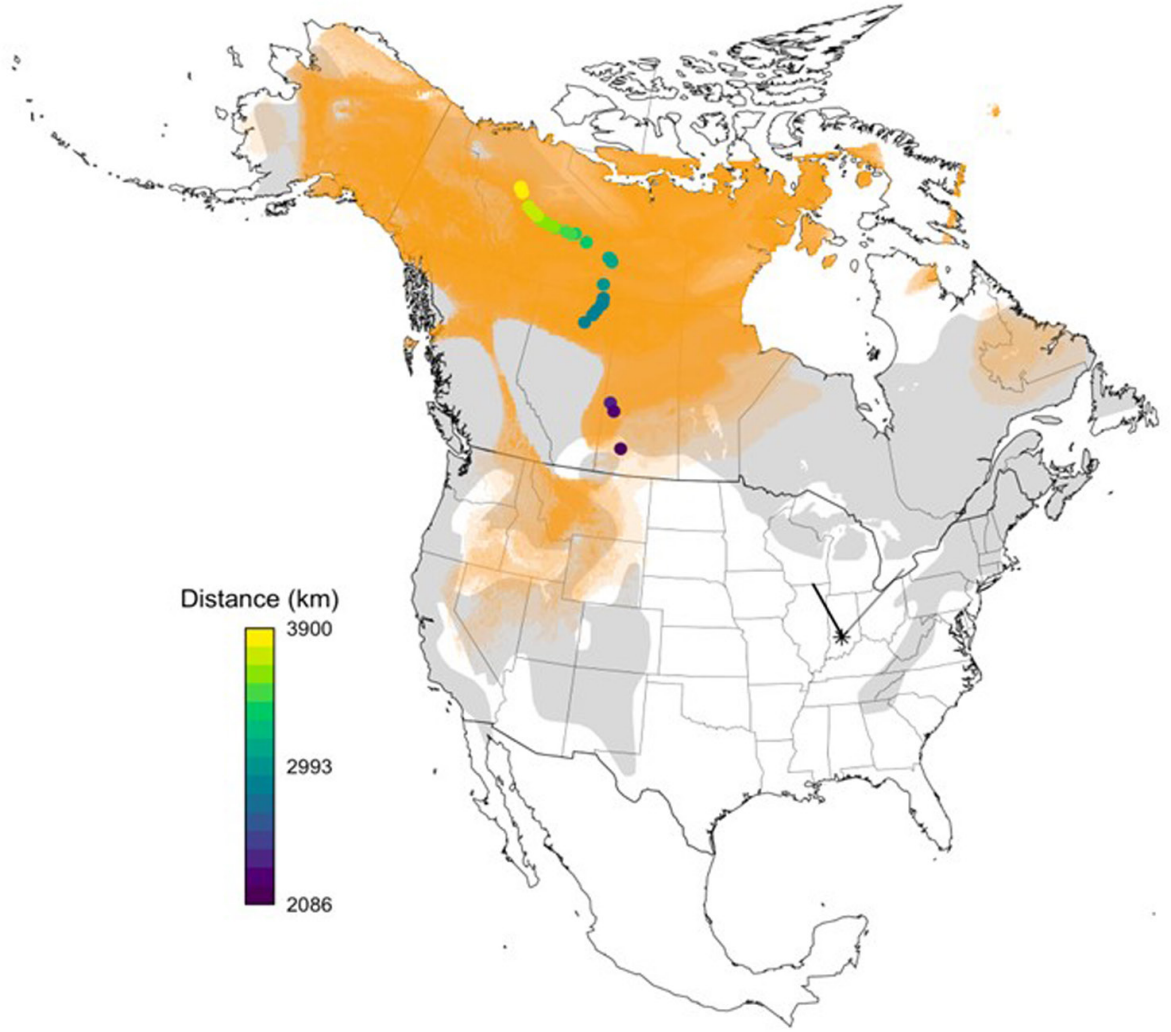
Estimated breeding locations and migratory distances based on junco feather δ^2^H. Orange shading represents overlapping geographic breeding assignments of individuals. Estimates reflect cells within the top 10% highest posterior probability (Bowen et al., 2014; Ma et al., 2020; Wunder, 2010). Points on the map represent the centroid of each estimated breeding polygon, colored by distance to location of capture (star; Bloomington, IN). Lines radiating from the star represent subsequent Motus detections after departure. The seasonal breeding range for *Junco hyemalis* (Baillie et al., 2004) is shown in light gray (the species' full range map was used to construct geographic assignment maps).

Hydrogen isotopic composition of junco feathers ranged from −158.9% to −95.8%, reflecting median breeding locations from 66 to 51° N, respectively (Figure 1). Maximum and minimum latitudinal estimates spanned from 70 to 36° N. Estimated distances to breeding grounds accordingly ranged from approximately 2086 to 3900 kilometers (x̄ = 3297, SE = 78.4). Incidentally, we detected six of our 37 tagged juncos at other Motus towers following their departure from Indiana (Figure 1). Five birds were detected at the Lake Petite station in Wisconsin (42.5117°, −88.5488°), and one bird was detected at the Werden station (42.7551°, −80.2724°) in Ontario, Canada.

### 3.2 Haemosporidian infection

We identified haemosporidian infections in 11 of 34 juncos prior to release (32.4%, 95% CI: 19.1–49.2%). Only two individuals had detectable *Haemoproteus* spp. infection (intensity = 16–23 infected erythrocytes from 100 fields of view), and nine individuals had detectable *Leucocytozoon* spp. infection (intensity = 1–7 infected erythrocytes from 100 fields of view); no individuals harbored co‐infecting haemosporidian parasites. We also opportunistically detected *Trypanosoma* spp. in a single bird (1/34, two parasites were detected from 100 fields of view), and this individual was not infected by either *Haemoproteus* spp. nor *Leucocytozoon* spp. parasites.

### 3.3 Extrinsic and intrinsic predictors of migration timing

**FIGURE 2:**
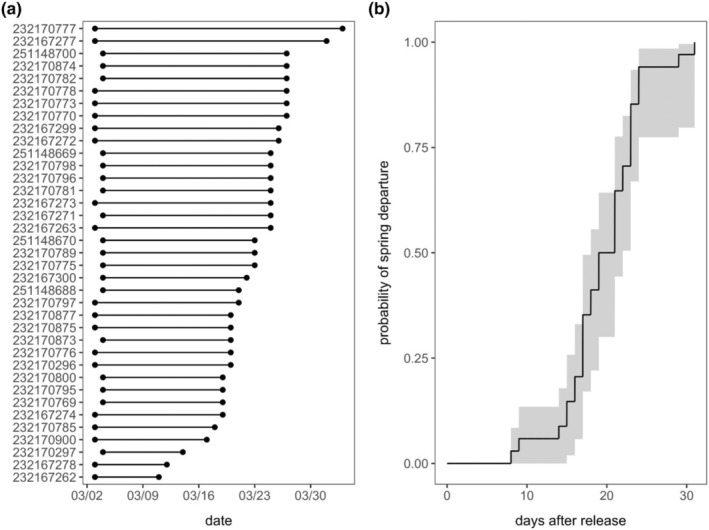
(a) Release and departure timing inferred from Motus at the Kent Farm Research Station for all 37 tagged juncos. (b) Mean probabilities of spring departure as inferred from Kaplan–Meier survival curves (i.e., 1—survival probabilities) using the *survival* R package alongside 95% confidence intervals.

All birds departed 8–31 days after release (x̄ = 19.11 ± 0.81 SE), between March 11 and April 3 2020 (Figure 2). Comparison among 10 CPHMMs predicting risk of departure date as a function of extrinsic and intrinsic factors identified only one competitive model, which included the nonlinear additive effects of wind speed and weather PC1 (*w*_*i*_ = 0.61; Table 1). Wind direction had the greatest relative importance among predictors (99%), followed by weather PC1 (62%); wind speed, the interaction between wind speed and weather PC1, and estimated migration distance all had lesser importance (38%, 22%, and 16%, respectively). All intrinsic predictors (i.e., fat, body condition, total leukocytes, HL ratios) and their interactions were unimportant (≤1%).

**TABLE 1:** Comparison of CPHMMs predicting junco risk of spring departure; all models include a random effect of site. Candidate models are ranked by ΔAICc alongside Akaike weights (*w*_*i*_) and deviance explained (DE).

Fixed effects	ΔAICc	*w*_*i*_	DE, %
~ s(wind direction) + s(weather PC1)	0.00	0.61	31
~ s(wind speed) + s(wind direction) + ti(wind speed, wind direction)	2.09	0.22	3
~ s(wind speed) + s(wind direction) + s(migration distance)	2.69	0.16	4
~ s(intensity) + s(weather PC1) + ti(intensity, weather PC1)	8.04	0.01	20
~ s(intensity) + s(HL ratios) + s(weather PC1)	12.71	<0.01	10
~ s(intensity) + s(total leukocytes) + ti(intensity, total leukocytes)	26.54	<0.01	25
~ s(body condition) + s(migration distance)	27.87	<0.01	5
~ s(fat score) + s(migration distance)	27.87	<0.01	5
~ s(intensity) + s(total leukocytes) + s(HL ratios)	28.15	<0.01	4
~ s(intensity) + s(HL ratios) + ti(intensity, HL ratios)	28.15	<0.01	4

**FIGURE 3:**
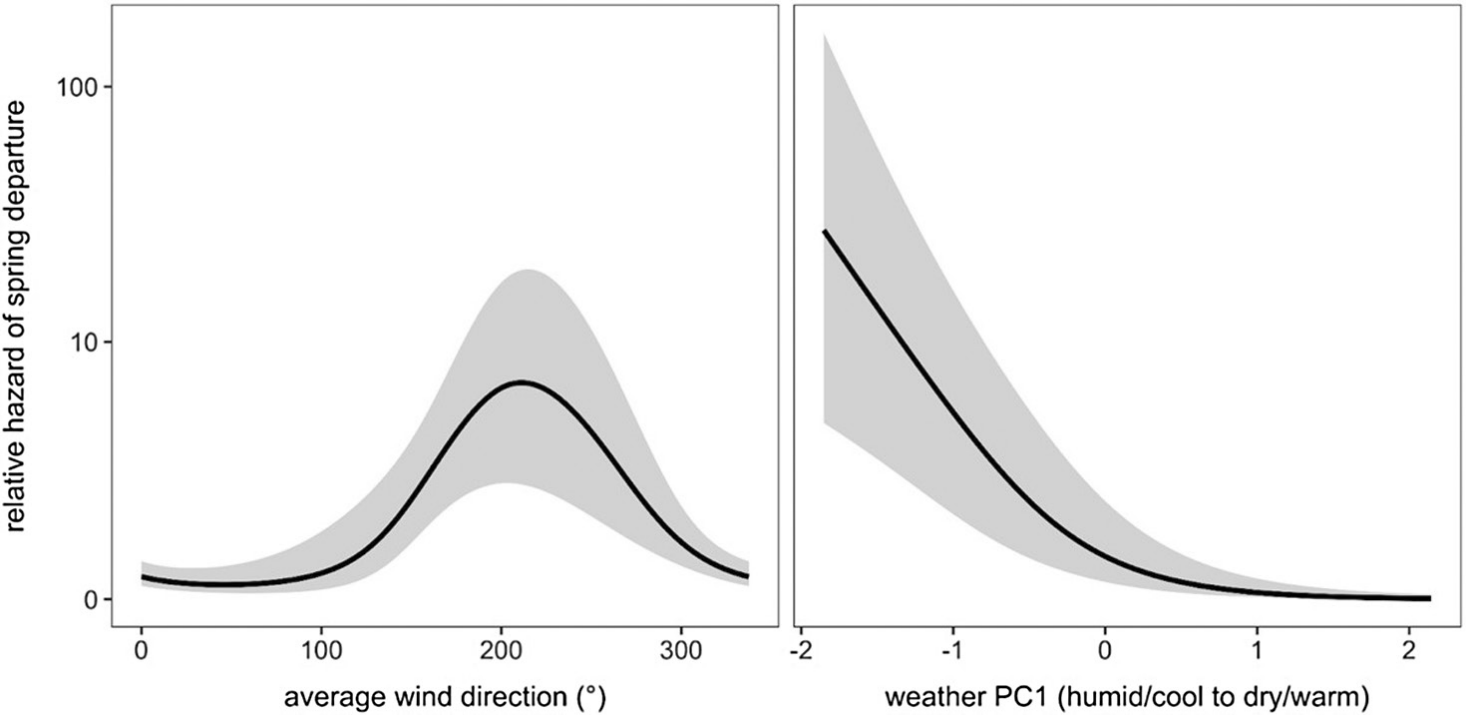
Relative hazards of spring departure estimated from the most competitive CPHMM as selected through AICc (*w*_*i*_ = 0.61). The predicted relative hazard and 95% confidence intervals from this model are displayed for mean wind direction and weather PC1.

Our top model explained 30.5% of the deviance in spring migration risk, with both wind direction ($\chi_{1.8,2}^{2}$ = 17.42, *p* < .001) and weather PC1 ($\chi_{1.4,3}^{2}$ = 29.61, *p* < .001) being significant nonlinear predictors of departure. Specifically, the risk of spring departure was greatest with average nightly southwestern winds and for humid and cool nights (Figure 3).

## 4 DISCUSSION

Numerous factors influence the timing of spring departure in birds, yet the relative influence of intrinsic and extrinsic variables on migration initiation remains a topic of debate. In this study, we tested the relative influence of these diverse factors on the risk of spring departure in a modestly sized group of wild‐caught juncos. Weather variables outperformed all tested intrinsic variables. We predicted that both wind direction and speed would have the greatest influence on departure; however, only wind direction was supported by our top model. Temperature and relative humidity (i.e., weather PC1) also influenced departures, with humid and cool nights having greater risk of departure than warmer, drier nights. While we predicted that birds with intense haemosporidian infection would depart later than uninfected individuals or those with low‐intensity infections, infection intensity did not influence departure risk. Additionally, higher fat reserves, lower leukocyte counts, and lower HL ratios did not affect departure risk. Our results suggest that the advantage of following winds outweigh the cost of haemosporidian infections and the other measured physiological variables during spring migration.

Many migratory birds harbor haemosporidian infections throughout the year, including during migration, (Cornelius et al., 2014; Pulgarín‐R et al., 2019; Ricklefs et al., 2017) and ongoing research continues to clarify the effects of avian malaria on migration timing, duration, and distance. Prior work on juncos found greater prevalence of haemosporidian infections in a non‐migratory subspecies compared with sympatric overwintering migrants following fall migration, suggesting that infected birds may be more likely to experience disease‐induced mortality during migration (Slowinski et al., 2018). Similarly, juncos with longer migrations were more likely to show elevated HL ratios upon arrival to their wintering grounds; this effect was stronger in haemosporidian‐infected birds, suggesting interactive effects of migration and infection on host energetics (Becker et al., 2019). Therefore, we predicted that juncos with more intense infections might delay migration initiation to mitigate these potential physiological costs. However, our results indicate that weather has a strong impact on migration irrespective of haemosporidian intensity. Importantly, our study focused on spring migration in adult juncos. While chronic haemosporidian infections may not impact migration timing during this life stage, it is unclear whether this pattern would hold true for juncos during autumn migration, especially juveniles. In fact, juvenile European robins (Erithacus rubecula) with haemosporidian infections arrive at wintering ground later than uninfected subadults (Ágh et al., 2019). Thus, season, age and stage of infection may influence whether haemosporidian infections delay migration initiation. Experimental infections in conjunction with departure tracking would provide more conclusive data on how weather interacts with parasite intensity to influence migration initiation.

Our analysis also indicates that migration distance and mean wind speed were relatively less informative predictors of spring departure date. Regarding migration distance, we expected that birds with longer spring migrations would depart earlier, as these individuals would potentially need to undertake prolonged stopovers to rest and refuel on the way to their breeding grounds. However, research suggests that long‐distance migrants have a greater ability to modify migratory behavior while en route and thus departure date may not be directly related to migration distance as migration speed and routes are flexible (La Sorte & Fink, 2017; Marra et al., 2005). While juncos have been well‐studied in regard to differential migration and migration timing (Cristol et al., 1999; Ketterson & Nolan, 1976), more research is essential to understanding how distance to breeding ground influences departure date. Isotopic feather analysis is advancing work in this field, as is the improvement and proliferation of nanotag technology (e.g., Motus, Cellular Tracking Technologies), which could help further distinguish the various influences on migration distance as it relates to departure.

Past studies have shown that both wind speed and direction are important predictors of departure and that tail winds can significantly increase flight times and greatly decrease energy requirements (Alerstam, 1979b; Bloch & Bruderer, 1982; Kemp et al., 2010; Liechti, 2006). There are several possibilities as to why these variables were less important in our analysis. Optimal wind selectivity is a product of many factors, including the size of the bird; rate of fat use; distance, duration, and altitude of migration; and even the wind pattern itself (Alerstam, 1979a). Lastly, birds here departed over a relatively short time frame (a minimum of 8 days after release, up to 31 days); therefore, there may not have been sufficient variation in wind speed and direction to outrank other predictors in our models.

In contrast to our expectations about physiological state and departure date, body condition, fat score, HL ratios, and leukocyte counts were also all uninformative predictors of spring migration date. Higher fat reserves are instrumental to migratory performance in songbirds (Price, 2010) and may promote the onset of migratory restlessness, advancing their departure (Lupi et al., 2017; Studds & Marra, 2005). The lack of a relationship between physiological measures and departure date observed here could reflect individual variation in migration strategies; for example, strong selective pressure to arrive early at the breeding grounds may motivate some birds to leave in relatively poor body condition and with high HL ratios and then compensate during stopover (Prop et al., 2003). Lastly, the time between release from captivity and the onset of spring migration (8–31 days) may have precluded a true representation of some physiological measures at departure; for example, maximum fat deposition rates in migratory songbirds have been reported as high as 12% differences in lean body mass in 1 day (Lindström, 1991).

The findings presented here add to the large body of information on the importance of weather on migration departure in songbirds. Importantly, our study focused on migratory departure in male juncos, while females may be differentially impacted by the variables measured. Indeed, this species shows sex‐based variation in overwintering latitudes, with females migrating earlier and further south than males (Nolan & Ketterson, 1990). Our sample size is a substantial limitation in understanding and asking these questions, as well. A more robust sample size would have increased the confidence in our findings. Additional data could also help understand whether the results of our study are context dependent; for example, intrinsic factors might become important during years with low food availability or inclement weather. This will likely be increasingly important for wildlife conservation going forward, as climate change is associated with increasingly severe temperatures and unpredictable weather events (Stott, 2016; Zhang et al., 2013).

## AUTHOR CONTRIBUTIONS

**Allison J. Byrd:** Conceptualization (equal); data curation (lead); methodology (equal); project administration (lead); supervision (lead); writing – original draft (lead); writing – review and editing (equal). **Katherine M. Talbott:** Conceptualization (equal); data curation (supporting); methodology (equal); writing – original draft (supporting); writing – review and editing (supporting). **Tara M. Smiley:** Formal analysis (equal); funding acquisition (supporting); investigation (supporting); methodology (supporting); writing – review and editing (supporting). **Taylor B. Verrett:** Data curation (supporting); investigation (supporting). **Michael S. Gross:** Investigation (supporting); validation (equal). **Michelle L. Hladik:** Investigation (supporting). **Ellen D. Ketterson:** Conceptualization (equal); funding acquisition (lead); methodology (equal); writing – review and editing (supporting). **Daniel J. Becker:** Conceptualization (equal); data curation (supporting); formal analysis (lead); funding acquisition (equal); investigation (equal); methodology (equal); writing – original draft (equal); writing – review and editing (equal).

## FUNDING INFORMATION

Funds were provided by Indiana University's Grand Challenge Initiative, Prepared for Environmental Change to EDK and TMS and from the University of Oklahoma to DJB.

## CONFLICT OF INTEREST STATEMENT

The authors declare they have no competing interests.

## DECLARATIONS

Any use of trade, firm, or product names is for descriptive purposes only and does not imply endorsement by the U.S. Government.

## Supporting information


Appendix S1.

## Data Availability

The data and R code that support the findings of this study are openly available in Dryad (https://doi.org/10.5061/dryad.msbcc2g3x).

## References

[ece310874-bib-2001] Able, K. P. (1973). The role of weather variables and flight direction in determining the magnitude of nocturnal bird migration. Ecology, 54(5), 1031–1041.

[ece310874-bib-0001] Ágh, N. , Piross, I. S. , Majoros, G. , Csörgő, T. , & Szöllősi, E. (2019). Malaria infection status of European Robins seems to associate with timing of autumn migration but not with actual condition. Parasitology, 146(6), 814–820. 30638174 10.1017/S0031182018002184

[ece310874-bib-0002] Alerstam, T. (1979a). Optimal use of wind by migrating birds: Combined drift and overcompensation. Journal of Theoretical Biology, 79(3), 341–353. 522498 10.1016/0022-5193(79)90351-5

[ece310874-bib-0003] Alerstam, T. (1979b). Wind as selective agent in bird migration. Ornis Scandinavica, 10(1), 76–93.

[ece310874-bib-0004] Asghar, M. , Hasselquist, D. , & Bensch, S. (2011). Are chronic avian haemosporidian infections costly in wild birds? Journal of Avian Biology, 42(6), 530–537.

[ece310874-bib-0005] Atkinson, C. T. , Woods, K. L. , Dusek, R. J. , Sileo, L. S. , & Iko, W. M. (1995). Wildlife disease and conservation in Hawaii: Pathogenicity of avian malaria (*Plasmodium relictum*) in experimentally infected iiwi (*Vestiaria coccinea*). Parasitology, 111(Suppl), S59–S69. 8632925 10.1017/s003118200007582x

[ece310874-bib-0006] Baillie, J. , Hilton‐Taylor, C. , Stuart, S. N. , & IUCN Species Survival Commission . (2004). 2004 IUCN red list of threatened species: A global species assessment. IUCN.

[ece310874-bib-0007] Bartoń, K. (2013). MuMIn: Multi‐Model Inference, version 1.9.0. *R Package*.

[ece310874-bib-0008] Becker, D. J. , Byrd, A. , Smiley, T. M. , Marques, M. F. , Nunez, J. V. , Talbott, K. M. , Atwell, J. W. , Volokhov, D. V. , Ketterson, E. D. , Jahn, A. E. , & Clark, K. L. (2022). Novel *Rickettsia* spp. in two common overwintering North American songbirds. Emerging Microbes & Infections, 11(1), 2746–2748. 36285426 10.1080/22221751.2022.2140610 PMC9662038

[ece310874-bib-0009] Becker, D. J. , Singh, D. , Pan, Q. , Montoure, J. D. , Talbott, K. M. , Wanamaker, S. M. , & Ketterson, E. D. (2020). Artificial light at night amplifies seasonal relapse of haemosporidian parasites in a widespread songbird. Proceedings. Biological Sciences/The Royal Society, 287(1935), 20201831. 10.1098/rspb.2020.1831 PMC7542808 32962545

[ece310874-bib-0010] Becker, D. J. , Talbott, K. M. , Smiley, T. M. , Clark, K. L. , Sauer, P. E. , & Ketterson, E. D. (2019). Leukocyte profiles vary with breeding latitude and infection status in a seasonally sympatric songbird. Animal Migration, 6(1), 28–40. 10.1515/ami-2019-0004

[ece310874-bib-0011] Bloch, R. , & Bruderer, B. (1982). The air speed of migrating birds and its relationship to the wind. Behavioral Ecology and Sociobiology, 11(1), 19–24.

[ece310874-bib-0012] Bowen, G. J. , Liu, Z. , & Vander Zanden, H. B. (2014). Geographic assignment with stable isotopes in IsoMAP. Methods in Ecology and Evolution/British Ecological Society, 5(3), 201–206. 10.1111/2041-210X.12147

[ece310874-bib-0013] Bowen, G. J. , & Revenaugh, J. (2003). Interpolating the isotopic composition of modern meteoric precipitation. Water Resources Research, 39(10). 10.1029/2003wr002086

[ece310874-bib-0014] Bowen, G. J. , Wassenaar, L. I. , & Hobson, K. A. (2005). Global application of stable hydrogen and oxygen isotopes to wildlife forensics. Oecologia, 143(3), 337–348. 15726429 10.1007/s00442-004-1813-y

[ece310874-bib-0015] Bridge, E. S. , Kelly, J. F. , Bjornen, P. E. , Curry, C. M. , Crawford, P. H. C. , & Paritte, J. M. (2010). Effects of nutritional condition on spring migration: Do migrants use resource availability to keep pace with a changing world? The Journal of Experimental Biology, 213(Pt 14), 2424–2429. 20581272 10.1242/jeb.041277

[ece310874-bib-0016] Brust, V. , Eikenaar, C. , Packmor, F. , Schmaljohann, H. , Hüppop, O. , & Czirják, G. Á. (2022). Do departure and flight route decisions correlate with immune parameters in migratory songbirds? Functional Ecology, 36(12), 3007–3021.

[ece310874-bib-0017] Burnham, K. P. , & Anderson, D. R. (2002). A practical information‐theoretic approach. In Model selection and multimodel inference (2nd ed.). Springer http://sutlib2.sut.ac.th/sut_contents/H79182.pdf

[ece310874-bib-0018] Campbell, T. W. (1995). Avian hematology and cytology. Journal of Zoo Animal Medicine, 19(4), 244.

[ece310874-bib-0019] Castro‐Santos, T. , & Haro, A. (2003). Quantifying migratory delay: A new application of survival analysis methods. Canadian Journal of Fisheries and Aquatic Sciences, 60(8), 986–996.

[ece310874-bib-0020] Chao, M. , Vander Zanden, H. B. , Wunder, M. B. , & Bowen, G. J. (2020). assignR: An r package for isotope‐based geographic assignment. Methods in Ecology and Evolution, 11(8), 996–1001.

[ece310874-bib-0021] Chapman, J. W. , Nilsson, C. , Lim, K. S. , Bäckman, J. , Reynolds, D. R. , & Alerstam, T. (2016). Adaptive strategies in nocturnally migrating insects and songbirds: Contrasting responses to wind. The Journal of Animal Ecology, 85(1), 115–124. 26147535 10.1111/1365-2656.12420

[ece310874-bib-0022] Cornelius, E. A. , Davis, A. K. , & Altizer, S. A. (2014). How important are hemoparasites to migratory songbirds? Evaluating physiological measures and infection status in three neotropical migrants during stopover. Physiological and Biochemical Zoology: PBZ, 87(5), 719–728. 25244383 10.1086/677541

[ece310874-bib-0023] Cosgrove, C. (2005). Avian malaria parasites and other haemosporidia—*Gediminas valkiunas*. 2004. CRC press, Boca Raton, Florida, USA. 932 pp. ISBN 0‐415‐30097‐5. US$170 (hardcover). Systematic Biology, 54(5), 860–863.

[ece310874-bib-0024] Covino, K. M. , Holberton, R. L. , & Morris, S. R. (2015). Factors influencing migratory decisions made by songbirds on spring stopover. Journal of Avian Biology, 46(1), 73–80.

[ece310874-bib-0025] Cristol, D. A. , Baker, M. B. , & Carbone, C. (1999). Differential Migration Revisited. In V. Nolan , E. D. Ketterson , & C. F. Thompson (Eds.), Current ornithology (pp. 33–88). Springer US.

[ece310874-bib-0026] Davis, A. K. , & Maney, D. L. (2018). The use of glucocorticoid hormones or leucocyte profiles to measure stress in vertebrates: What's the difference? Methods in Ecology and Evolution/British Ecological Society, 9(6), 1556–1568.

[ece310874-bib-0027] Davis, A. K. , Maney, D. L. , & Maerz, J. C. (2008). The use of leukocyte profiles to measure stress in vertebrates: A review for ecologists. Functional Ecology, 22(5), 760–772.

[ece310874-bib-0028] de Angeli Dutra, D. , Fecchio, A. , Braga, É. M. , & Poulin, R. (2022). Migratory behaviour does not alter cophylogenetic congruence between avian hosts and their haemosporidian parasites. Parasitology, 149(7), 905–912. 10.1017/S0031182022000154 PMC10090587 35393002

[ece310874-bib-0029] Deviche, P. , Greiner, E. C. , & Manteca, X. (2001). Seasonal and age‐related changes in blood parasite prevalence in Dark‐eyed Juncos (*Junco hyemalis*, Aves, Passeriformes). The Journal of Experimental Zoology, 289(7), 456–466. 11351333 10.1002/jez.1027

[ece310874-bib-0030] Diamond, H. J. , Karl, T. R. , Palecki, M. A. , Bruce Baker, C. , Bell, J. E. , Leeper, R. D. , Easterling, D. R. , Lawrimore, J. H. , Meyers, T. P. , Helfert, M. R. , Goodge, G. , & Thorne, P. W. (2013). U.S. Climate reference network after one decade of operations: Status and assessment. Bulletin of the American Meteorological Society, 94(4), 485–498.

[ece310874-bib-0031] Dietsch, T. V. (2005). Seasonal variation of infestation by ectoparasitic chigger mite larvae (Acarina: Trombiculidae) on resident and migratory birds in coffee agroecosystems of Chiapas, Mexico. The Journal of Parasitology, 91(6), 1294–1303. 16539008 10.1645/GE-558R.1

[ece310874-bib-0032] Dossman, B. C. , Mitchell, G. W. , Norris, D. R. , Taylor, P. D. , Guglielmo, C. G. , Matthews, S. N. , & Rodewald, P. G. (2015). The effects of wind and fuel stores on stopover departure behavior across a migratory barrier. Behavioral Ecology: Official Journal of the International Society for Behavioral Ecology, 27(2), 567–574.

[ece310874-bib-0033] Drake, A. , Rock, C. A. , Quinlan, S. P. , Martin, M. , & Green, D. J. (2014). Wind speed during migration influences the survival, timing of breeding, and productivity of a neotropical migrant, *Setophaga petechia* . PLoS One, 9(5), e97152. 24828427 10.1371/journal.pone.0097152 PMC4020938

[ece310874-bib-0034] Eikenaar, C. , Fritzsch, A. , & Bairlein, F. (2013). Corticosterone and migratory fueling in Northern wheatears facing different barrier crossings. General and Comparative Endocrinology, 186, 181–186. 23518480 10.1016/j.ygcen.2013.02.042

[ece310874-bib-0035] Eikenaar, C. , Müller, F. , & Leutgeb, C. (2017). Corticosterone and timing of migratory departure in a songbird. Proceedings of the Royal Society B: Biological Sciences, 284(1846), 20162300. 10.1098/rspb.2016.2300 PMC5247501 28077768

[ece310874-bib-0036] Eng, M. L. , Stutchbury, B. J. M. , & Morrissey, C. A. (2017). Imidacloprid and chlorpyrifos insecticides impair migratory ability in a seed‐eating songbird. Scientific Reports, 7(1), 15176. 29123163 10.1038/s41598-017-15446-x PMC5680183

[ece310874-bib-0037] Erni, B. , Liechti, F. , Underhill, L. G. , & Bruderer, B. (2002). Wind and rain govern the intensity of nocturnal bird migration in Central Europe—A log‐linear regression analysis. Ardea, 90(1), 155–166.

[ece310874-bib-0038] Ferrer, M. M. (2022). Avian haemosporidian blood parasite diversity, prevalence, and distribution in Michigan's Western Upper Peninsula (Doctoral dissertation, Michigan Technological University). 10.37099/mtu.dc.etdr/1470

[ece310874-bib-0039] Fudickar, A. M. , Greives, T. J. , Atwell, J. W. , Stricker, C. A. , & Ketterson, E. D. (2016). Reproductive allochrony in seasonally sympatric populations maintained by differential response to photoperiod: Implications for population divergence and response to climate change. The American Naturalist, 187(4), 436–446. 10.1086/685296 27028072

[ece310874-bib-0040] Garvin, M. C. , Szell, C. C. , & Moore, F. R. (2006). Blood parasites of nearctic–neotropical migrant passerine birds during spring trans‐gulf migration: Impact on host body condition. The Journal of Parasitology, 92(5), 990–996. 17152939 10.1645/GE-758R.1

[ece310874-bib-0041] Griffiths, R. , Double, M. C. , Orr, K. , & Dawson, R. J. (1998). A DNA test to sex most birds. Molecular Ecology, 7(8), 1071–1075. 9711866 10.1046/j.1365-294x.1998.00389.x

[ece310874-bib-0042] Gunnarsson, T. G. , Gill, J. A. , Atkinson, P. W. , Gélinaud, G. , Potts, P. M. , Croger, R. E. , Gudmundsson, G. A. , Appleton, G. F. , & Sutherland, W. J. (2006). Population‐scale drivers of individual arrival times in migratory birds. The Journal of Animal Ecology, 75(5), 1119–1127. 16922847 10.1111/j.1365-2656.2006.01131.x

[ece310874-bib-0043] Hebrard, J. J. (1971). The nightly initiation of passerine migration in spring: A direct visual study. The Ibis, 113(1), 8–18. 10.1111/j.1474-919X.1971.tb05119.x

[ece310874-bib-0044] Hijmans, R. J. (2019). Introduction to the ‘raster’ package (version 2.8‐19) . https://mran.microsoft.com/snapshot/2015‐08‐12/web/packages/raster/vignettes/Raster.pdf

[ece310874-bib-0045] Hobson, K. A. (1999). Tracing origins and migration of wildlife using stable isotopes: A review. Oecologia, 120(3), 314–326. 28308009 10.1007/s004420050865

[ece310874-bib-0046] Hobson, K. A. , Van Wilgenburg, S. L. , Wassenaar, L. I. , & Larson, K. (2012). Linking hydrogen (δ2H) isotopes in feathers and precipitation: Sources of variance and consequences for assignment to isoscapes. PLoS One, 7(4), e35137. 22509393 10.1371/journal.pone.0035137 PMC3324428

[ece310874-bib-0047] Holberton, R. L. (1999). Changes in patterns of corticosterone secretion concurrent with migratory fattening in a neotropical migratory bird. General and Comparative Endocrinology, 116(1), 49–58. 10525361 10.1006/gcen.1999.7336

[ece310874-bib-0048] Horton, K. G. , Van Doren, B. M. , Stepanian, P. M. , Farnsworth, A. , & Kelly, J. F. (2016). Seasonal differences in landbird migration strategies. The Auk, 133(4), 761–769.

[ece310874-bib-0049] Hüppop, O. , & Winkel, W. (2006). Climate change and timing of spring migration in the long‐distance migrant *Ficedula hypoleuca* in central Europe: The role of spatially different temperature changes along migration routes. Journal of Ornithology/DO‐G, 147(2), 344–353.

[ece310874-bib-0050] Hurlbert, A. H. , & Liang, Z. (2012). Spatiotemporal variation in avian migration phenology: Citizen science reveals effects of climate change. PLoS One, 7(2), e31662. 22384050 10.1371/journal.pone.0031662 PMC3285173

[ece310874-bib-0051] Jawor, J. M. , McGlothlin, J. W. , Casto, J. M. , Greives, T. J. , Snajdr, E. A. , Bentley, G. E. , & Ketterson, E. D. (2006). Seasonal and individual variation in response to GnRH challenge in male dark‐eyed juncos (*Junco hyemalis*). General and Comparative Endocrinology, 149(2), 182–189. 16814785 10.1016/j.ygcen.2006.05.013

[ece310874-bib-0052] Kelly, T. R. , MacGillivray, H. L. , Sarquis‐Adamson, Y. , Watson, M. J. , Hobson, K. A. , & MacDougall‐Shackleton, E. A. (2016). Seasonal migration distance varies with natal dispersal and predicts parasitic infection in song sparrows. Behavioral Ecology and Sociobiology, 70(11), 1857–1866. 10.1007/s00265-016-2191-2

[ece310874-bib-0053] Kelly, T. R. , Rubin, B. D. , MacDougall‐Shackleton, S. A. , & MacDougall‐Shackleton, E. A. (2020). Experimental malaria infection affects songbirds' nocturnal migratory activity. Physiological and Biochemical Zoology: PBZ, 93(2), 97–110. 32013740 10.1086/707495

[ece310874-bib-0054] Kemp, M. U. , Shamoun‐Baranes, J. , Van Gasteren, H. , Bouten, W. , & Van Loon, E. E. (2010). Can wind help explain seasonal differences in avian migration speed? Journal of Avian Biology, 41(6), 672–677.

[ece310874-bib-0055] Ketterson, E. D. , & Nolan, V., Jr. (1976). Geographic variation and its climatic correlates in the sex ratio of eastern‐wintering dark‐eyed juncos (*Junco hyemalis hyemalis*). Ecology, 57(4), 679–693.

[ece310874-bib-0056] Ketterson, E. D. , & Nolan, V. (1982). The role of migration and winter mortality in the life history of a temperate‐zone migrant, the Dark‐eyed Junco, as determined from demographic analyses of winter populations. The Auk, 99(2), 243–259.

[ece310874-bib-0057] Klaassen, M. , Hoye, B. J. , Nolet, B. A. , & Buttemer, W. A. (2012). Ecophysiology of avian migration in the face of current global hazards. Philosophical Transactions of the Royal Society of London. Series B, Biological Sciences, 367(1596), 1719–1732. 22566678 10.1098/rstb.2012.0008 PMC3350656

[ece310874-bib-0058] Kubelka, V. , Sandercock, B. K. , Székely, T. , & Freckleton, R. P. (2022). Animal migration to northern latitudes: Environmental changes and increasing threats. Trends in Ecology & Evolution, 37(1), 30–41. 34579979 10.1016/j.tree.2021.08.010

[ece310874-bib-0059] La Sorte, F. A. , & Fink, D. (2017). Migration distance, ecological barriers and en‐route variation in the migratory behaviour of terrestrial bird populations. Global Ecology and Biogeography: A Journal of Macroecology, 26(2), 216–227.

[ece310874-bib-0060] Labocha, M. K. , & Hayes, J. P. (2012). Morphometric indices of body condition in birds: A review. Journal of Ornithology/DO‐G, 153(1), 1–22.

[ece310874-bib-0061] Lack, D. (1963). Weather factors initiating migration. In: *Proceedings XIII International Ornithological Congress*.

[ece310874-bib-0062] Lack, D. , & Eastwood, E. (1962). Radar films of migration over eastern England. British Birds; An Illustrated Magazine Devoted to the Birds on the British List, 55(9), 388–414.

[ece310874-bib-0063] Landys, M. M. , Wingfield, J. C. , & Ramenofsky, M. (2004). Plasma corticosterone increases during migratory restlessness in the captive white‐crowned sparrow *Zonotrichia leucophrys gambelli* . Hormones and Behavior, 46(5), 574–581. 15555499 10.1016/j.yhbeh.2004.06.006

[ece310874-bib-0064] Lawrence, M. G. (2005). The relationship between relative humidity and the dewpoint temperature in moist air: A simple conversion and applications. Bulletin of the American Meteorological Society, 86(2), 225–234.

[ece310874-bib-0065] Liechti, F. (2006). Birds: Blowin' by the wind? Journal of Ornithology/DO‐G, 147(2), 202–211.

[ece310874-bib-0066] Lindström, Å. (1991). Maximum fat deposition rates in migrating birds. Ornis Scandinavica, 22(1), 12–19.

[ece310874-bib-0067] Lochmiller, R. L. , & Deerenberg, C. (2000). Trade‐offs in evolutionary immunology: Just what is the cost of immunity? Oikos, 88(1), 87–98.

[ece310874-bib-0068] Lupi, S. , Maggini, I. , Goymann, W. , Cardinale, M. , Rojas Mora, A. , & Fusani, L. (2017). Effects of body condition and food intake on stop‐over decisions in Garden Warblers and European Robins during spring migration. Journal of Ornithology/DO‐G, 158(4), 989–999.

[ece310874-bib-0169] Ma, C. , Vander Zanden, H. B. , Wunder, M. B. , & Bowen, G. J. (2020). assignR: An R package for isotope‐based geographic assignment. Methods in Ecology and Evolution/British Ecological Society, 11(8), 996–1001.

[ece310874-bib-0069] Marra, P. P. , Francis, C. M. , Mulvihill, R. S. , & Moore, F. R. (2005). The influence of climate on the timing and rate of spring bird migration. Oecologia, 142(2), 307–315. 15480801 10.1007/s00442-004-1725-x

[ece310874-bib-0070] Martínez‐Renau, E. , Rojas‐Estévez, N. , Friis, G. , Hernández‐Montoya, J. C. , Elizondo, P. , & Milá, B. (2022). Haemosporidian parasite diversity and prevalence in the songbird genus *Junco* across Central and North America. Ornithology, 139(3), ukac022.

[ece310874-bib-0071] Merrill, L. , Levengood, J. M. , England, J. C. , Osborn, J. M. , & Hagy, H. M. (2018). Blood parasite infection linked to condition of spring‐migrating Lesser Scaup (*Aythya affinis*). Canadian Journal of Zoology, 96(10), 1145–1152.

[ece310874-bib-0072] Møller, A. P. (1994). Phenotype‐dependent arrival time and its consequences in a migratory bird. Behavioral Ecology and Sociobiology, 35(2), 115–122.

[ece310874-bib-0073] Mukhin, A. , Palinauskas, V. , Platonova, E. , Kobylkov, D. , Vakoliuk, I. , & Valkiūnas, G. (2016). The strategy to survive primary malaria infection: An experimental study on behavioural changes in parasitized birds. PLoS One, 11(7), e0159216. 27434058 10.1371/journal.pone.0159216 PMC4951008

[ece310874-bib-0074] Nakagawa, S. , & Schielzeth, H. (2013). A general and simple method for obtaining R^2^ from generalized linear mixed‐effects models. Methods in Ecology and Evolution/British Ecological Society, 4(2), 133–142.

[ece310874-bib-0075] Newton, I. (2010). The migration ecology of birds. Elsevier.

[ece310874-bib-0076] Nolan, V., Jr. , & Ketterson, E. D. (1990). Timing of autumn migration and its relation to winter distribution in dark‐eyed juncos. Ecology, 71(4), 1267–1278.

[ece310874-bib-0077] Nussbaumer, R. , Schmid, B. , Bauer, S. , & Liechti, F. (2022). Favorable winds speed up bird migration in spring but not in autumn. Ecology and Evolution, 12(8), e9146. 35923938 10.1002/ece3.9146 PMC9339755

[ece310874-bib-0078] Owen, J. C. , & Moore, F. R. (2008). Relationship between energetic condition and indicators of immune function in thrushes during spring migration. Canadian Journal of Zoology, 86(7), 638–647.

[ece310874-bib-0079] Price, E. R. (2010). Dietary lipid composition and avian migratory flight performance: Development of a theoretical framework for avian fat storage. Comparative Biochemistry and Physiology. Part A, Molecular & Integrative Physiology, 157(4), 297–309. 10.1016/j.cbpa.2010.05.019 20561892

[ece310874-bib-0080] Prop, J. , Black, J. M. , & Shimmings, P. (2003). Travel schedules to the high arctic: Barnacle geese trade‐off the timing of migration with accumulation of fat deposits. Oikos, 103(2), 403–414.

[ece310874-bib-0081] Pulgarín‐R, P. C. , Gómez, C. , Bayly, N. J. , Bensch, S. , FitzGerald, A. M. , Starkloff, N. , Kirchman, J. J. , González‐Prieto, A. M. , Hobson, K. A. , Ungvari‐Martin, J. , Skeen, H. , Castaño, M. I. , & Cadena, C. D. (2019). Migratory birds as vehicles for parasite dispersal? Infection by avian haemosporidians over the year and throughout the range of a long‐distance migrant. Journal of Biogeography, 46(1), 83–96.

[ece310874-bib-0083] Ramenofsky, M. (1990). Fat storage and fat metabolism in relation to migration (pp. 214–231). *Physiology and Ecophysiology* .

[ece310874-bib-0084] Reed, K. D. , Meece, J. K. , Henkel, J. S. , & Shukla, S. K. (2003). Birds, migration and emerging zoonoses: West Nile virus, lyme disease, influenza A and enteropathogens. Clinical Medicine & Research, 1(1), 5–12. 15931279 10.3121/cmr.1.1.5 PMC1069015

[ece310874-bib-0085] Richardson, W. J. (1978). Timing and amount of bird migration in relation to weather: A Review. Oikos, 30(2), 224–272.

[ece310874-bib-0086] Ricklefs, R. E. , Medeiros, M. , Ellis, V. A. , Svensson‐Coelho, M. , Blake, J. G. , Loiselle, B. A. , Soares, L. , Fecchio, A. , Outlaw, D. , Marra, P. P. , Latta, S. C. , Valkiūnas, G. , Hellgren, O. , & Bensch, S. (2017). Avian migration and the distribution of malaria parasites in New World passerine birds. Journal of Biogeography, 44(5), 1113–1123.

[ece310874-bib-0087] Rotics, S. , Kaatz, M. , Turjeman, S. , Zurell, D. , Wikelski, M. , Sapir, N. , Eggers, U. , Fiedler, W. , Jeltsch, F. , & Nathan, R. (2018). Early arrival at breeding grounds: Causes, costs and a trade‐off with overwintering latitude. The Journal of Animal Ecology, 87(6), 1627–1638. 30120893 10.1111/1365-2656.12898

[ece310874-bib-0088] Rubenstein, D. R. , & Hobson, K. A. (2004). From birds to butterflies: Animal movement patterns and stable isotopes. Trends in Ecology & Evolution, 19(5), 256–263. 16701265 10.1016/j.tree.2004.03.017

[ece310874-bib-0089] Saino, N. , Rubolini, D. , Jonzén, N. , Ergon, T. , Montemaggiori, A. , Stenseth, N. C. , & Spina, F. (2007). Temperature and rainfall anomalies in Africa predict timing of spring migration in trans‐Saharan migratory birds. Climate Research, 35, 123–134.

[ece310874-bib-0090] Santiago‐Alarcon, D. , Mettler, R. , Segelbacher, G. , & Schaefer, H. M. (2013). Haemosporidian parasitism in the blackcap *Sylvia atricapillain* relation to spring arrival and body condition. Journal of Avian Biology, 44(6), 521–530.

[ece310874-bib-0091] Satterfield, D. A. , Marra, P. P. , Sillett, T. S. , & Altizer, S. (2018). Responses of migratory species and their pathogens to supplemental feeding. Philosophical Transactions of the Royal Society of London. Series B, Biological Sciences, 373(1745), 20170094. 10.1098/rstb.2017.0094 29531149 PMC5883000

[ece310874-bib-0092] Sauer, P. E. , Schimmelmann, A. , Sessions, A. L. , & Topalov, K. (2009). Simplified batch equilibration for D/H determination of non‐exchangeable hydrogen in solid organic material. Rapid Communications in Mass Spectrometry: RCM, 23(7), 949–956. 19241415 10.1002/rcm.3954

[ece310874-bib-0093] Schimmelmann, A. (1991). Determination of the concentration and stable isotopic composition of nonexchangeable hydrogen in organic matter. Analytical Chemistry, 63(21), 2456–2459.

[ece310874-bib-0094] Schimmelmann, A. , Lewan, M. D. , & Wintsch, R. P. (1999). D/H isotope ratios of kerogen, bitumen, oil, and water in hydrous pyrolysis of source rocks containing kerogen types I, II, IIS, and III. Geochimica et Cosmochimica Acta, 63(22), 3751–3766.

[ece310874-bib-0095] Schmaljohann, H. , Liechti, F. , & Bruderer, B. (2009). Trans‐Sahara migrants select flight altitudes to minimize energy costs rather than water loss. Behavioral Ecology and Sociobiology, 63(11), 1609–1619.

[ece310874-bib-0096] Schulte‐Hostedde, A. I. , Zinner, B. , Millar, J. S. , & Hickling, G. J. (2005). Restitution of mass–size residuals: Validating body condition indices. Ecology, 86(1), 155–163.

[ece310874-bib-0097] Serra‐Cobo, J. , Sanz‐Trullén, V. , & Martínez‐Rica, J. P. (1998). Migratory movements of *Miniopterus schreibersii* in the north‐east of Spain. Acta Theriologica, 43(3), 271–283.

[ece310874-bib-0098] Sinelschikova, A. , Kosarev, V. , Panov, I. , & Baushev, A. N. (2007). The influence of wind conditions in Europe on the advance in timing of the spring migration of the song thrush (*Turdus philomelos*) in the south‐east Baltic region. International Journal of Biometeorology, 51(5), 431–440. 17262220 10.1007/s00484-006-0077-0

[ece310874-bib-0099] Singh, D. , Reed, S. R. , Kimmitt, A. A. , Alford, K. A. , & Ketterson, E. D. (2019). Breeding at higher latitude as measured by stable isotope is associated with higher photoperiod threshold and delayed reproductive development in a songbird. bioRxiv, 789008. 10.1101/789008 33259797

[ece310874-bib-0100] Slowinski, S. P. , Fudickar, A. M. , Hughes, A. M. , Mettler, R. D. , Gorbatenko, O. V. , Spellman, G. M. , Ketterson, E. D. , & Atwell, J. W. (2018). Sedentary songbirds maintain higher prevalence of haemosporidian parasite infections than migratory conspecifics during seasonal sympatry. PLoS One, 13(8), e0201563. 30133475 10.1371/journal.pone.0201563 PMC6104930

[ece310874-bib-0101] Smith, R. J. , & Moore, F. R. (2005). Arrival timing and seasonal reproductive performance in a long‐distance migratory landbird. Behavioral Ecology and Sociobiology, 57(3), 231–239.

[ece310874-bib-0102] Stott, P. (2016). How climate change affects extreme weather events. Science, 352(6293), 1517–1518. 27339968 10.1126/science.aaf7271

[ece310874-bib-0103] Studds, C. E. , & Marra, P. P. (2005). Nonbreeding habitat occupancy and population processes: An upgrade experiment with a migratory bird. Ecology, 86(9), 2380–2385.

[ece310874-bib-0104] Talbott, K. M. , Becker, D. J. , Soini, H. A. , Higgins, B. J. , Novotny, M. V. , & Ketterson, E. D. (2022). Songbird preen oil odour reflects haemosporidian parasite load. Animal Behaviour, 188, 147–155. 35756157 10.1016/j.anbehav.2022.04.004 PMC9223275

[ece310874-bib-0105] Taylor, P. , Crewe, T. , Mackenzie, S. , Lepage, D. , Aubry, Y. , Crysler, Z. , Finney, G. , Francis, C. , Guglielmo, C. , Hamilton, D. , Holberton, R. L. , Loring, P. H. , Mitchell, G. W. , Norris, D. R. , Paquet, J. , Ronconi, R. A. , Smetzer, J. R. , Smith, P. A. , Welch, L. J. , & Woodworth, B. K. (2017). The Motus Wildlife Tracking System: A collaborative research network to enhance the understanding of wildlife movement. Avian Conservation and Ecology/Ecologie et Conservation Des Oiseaux, 12(1), 8. https://www.ace‐eco.org/vol12/iss1/art8/

[ece310874-bib-0106] Therneau, T. M. , & Grambsch, P. M. (2000). Modeling survival sata: Extending the Cox model. Springer US.

[ece310874-bib-0107] Thorsen, S. (1995–2023). Past weather in Bloomington, Indiana, USA—March 2020 . Time and Date. https://www.timeanddate.com/

[ece310874-bib-0108] Tøttrup, A. P. , Rainio, K. , Coppack, T. , Lehikoinen, E. , Rahbek, C. , & Thorup, K. (2010). Local temperature fine‐tunes the timing of spring migration in birds. Integrative and Comparative Biology, 50(3), 293–304. 21558204 10.1093/icb/icq028

[ece310874-bib-0109] Usui, T. , Butchart, S. H. M. , & Phillimore, A. B. (2017). Temporal shifts and temperature sensitivity of avian spring migratory phenology: A phylogenetic meta‐analysis. The Journal of Animal Ecology, 86(2), 250–261. 27859281 10.1111/1365-2656.12612 PMC6849580

[ece310874-bib-0110] Valkiunas, G. , Iezhova, T. A. , Krizanauskiene, A. , Palinauskas, V. , Sehgal, R. N. M. , & Bensch, S. (2008). A comparative analysis of microscopy and PCR‐based detection methods for blood parasites. The Journal of Parasitology, 94(6), 1395–1401. 18576856 10.1645/GE-1570.1

[ece310874-bib-0111] Van Doren, B. M. , & Horton, K. G. (2018). A continental system for forecasting bird migration. Science, 361(6407), 1115–1118. 30213913 10.1126/science.aat7526

[ece310874-bib-0112] Visser, M. E. , Perdeck, A. C. , Van Balen, J. H. , & Both, C. (2009). Climate change leads to decreasing bird migration distances. Global Change Biology, 15(8), 1859–1865.

[ece310874-bib-0113] Wanamaker, S. M. , Singh, D. , Byrd, A. J. , Smiley, T. M. , & Ketterson, E. D. (2020). Local adaptation from afar: Migratory bird populations diverge in the initiation of reproductive timing while wintering in sympatry. Biology Letters, 16(10), 20200493. 33023381 10.1098/rsbl.2020.0493 PMC7655480

[ece310874-bib-0114] Weber, T. P. , Houston, A. I. , & Ens, B. J. (1994). Optimal departure fat loads and stopover site use in avian migration: An analytical model. Proceedings. Biological Sciences/The Royal Society, 258(1351), 29–34.

[ece310874-bib-0115] Wikelski, M. , Tarlow, E. M. , Raim, A. , Diehl, R. H. , Larkin, R. P. , & Visser, G. H. (2003). Costs of migration in free‐flying songbirds. Nature, 423(6941), 704. 12802324 10.1038/423704a

[ece310874-bib-0116] Wilcove, D. S. , & Wikelski, M. (2008). Going, going, gone: Is animal migration disappearing. PLoS Biology, 6(7), e188. 18666834 10.1371/journal.pbio.0060188 PMC2486312

[ece310874-bib-0117] Witter, M. S. , & Cuthill, I. C. (1993). The ecological costs of avian fat storage. Philosophical Transactions of the Royal Society of London. Series B, Biological Sciences, 340(1291), 73–92. 8099746 10.1098/rstb.1993.0050

[ece310874-bib-0118] Wood, S. N. (2017). Generalized Additive Models: An Introduction with R (2nd ed.). CRC Press.

[ece310874-bib-0119] Wunder, M. B. (2010). Using isoscapes to model probability surfaces for determining geographic origins. In J. B. West , G. J. Bowen , T. E. Dawson , & K. P. Tu (Eds.), Isoscapes (pp. 251–270). Springer. https://link.springer.com/chapter/10.1007/978‐90‐481‐3354‐3_12

[ece310874-bib-0120] Yorinks, N. , & Atkinson, C. T. (2000). Effects of malaria on activity budgets of experimentally infected Juvenile Apapane (*Himatione sanguinea*). The Auk, 117(3), 731–738.

[ece310874-bib-0121] Zhang, J. , & Wu, L. (2018). The influence of population movements on the urban relative humidity of Beijing during the Chinese Spring Festival holiday. Journal of Cleaner Production, 170, 1508–1513.

[ece310874-bib-0122] Zhang, X. , Wan, H. , Zwiers, F. W. , Hegerl, G. C. , & Min, S.‐K. (2013). Attributing intensification of precipitation extremes to human influence. Geophysical Research Letters, 40(19), 5252–5257.

