# Supplementary material for: Determinants of spring migration departure dates in a New World sparrow: Weather variables reign supreme
Source: Ecol Evol. 2024 Feb 22;14(2):e10874. doi: 10.1002/ece3.10874 (PMC10883105; doi:10.1002/ece3.10874)
Supplement: Supplementary file 1 — Appendix S1. [file ECE3-14-e10874-s001.docx]

**Supplementary Material**

**Determinants of Spring Migration Departure Dates in a New World Sparrow: Weather Variables Reign Supreme**

Allison J. Byrd^1,2^*, Katherine M. Talbott^2^, Tara M. Smiley^3^, Taylor B. Verrett^5^, Michael S. Gross^4^, Michelle L. Hladik^4^, Ellen D. Ketterson^1,2^, Daniel J. Becker^5^*

^1^Environmental Resilience Institute, Indiana University, Bloomington, IN

^2^Department of Biology, Indiana University, Bloomington, IN

^3^Department of Ecology and Evolution, Stony Brook University, Stony Brook, NY

^4^U.S. Geological Survey, California Water Science Center, Sacramento, CA, 95819

^5^School of Biological Sciences, University of Oklahoma, Norman, OK

**Corresponding author**: Allison Byrd, e-mail: abyrd.in.uni@gmail.com, present address: Biology Building, 1001 E 3rd St, Bloomington, IN 47405

**Disclaimer:** Any use of trade, firm, or product names is for descriptive purposes only and does not imply endorsement by the U.S. Government.

Appendix A: Sample preparation for imidacloprid testing

*Dosing solution*

The imidacloprid dosing solution was prepared in organic sunflower oil. To confirm the concentration of imidacloprid in the dosing solution, the solution was first diluted 1:100 in acetone, followed by a sequential 1:100 dilution in 50/50 (v/v) acetonitrile/water. The solution was spiked with a clothianidin-d_3_ internal standard solution and analyzed by LC-MS/MS.

*Plasma samples*

Plasma volumes were measured using a 100 µL syringe and ranged 4-80 µL. Samples were spiked with an imidacloprid-d_4_ surrogate solution to monitor recovery. Plasma was precipitated by the addition of 350 µL of 0.5% formic acid in acetonitrile and cleaned-up via pass-through solid-phase extraction using Captiva EMR-Lipid cartridges (Agilent Technologies; 3 cc, 300 mg). Samples were evaporated to 100 µL under nitrogen and spiked with clothianidin-d_3_ internal standard prior to LC-MS/MS analysis.

*Seed samples*

Bird seed samples were massed to approximately 2.5 g and fortified with imidacloprid-d_4_ surrogate solution. Samples were extracted 5 mL of 50/50 (v/v) acetone/dichloromethane via sonication for 15 min. The extraction was repeated with 5 mL of fresh solvent. Seed samples were evaporated under nitrogen, solvent exchanged into 50/50 (v/v) acetonitrile/water and filtered to remove particulates. Samples spiked with clothianidin-d_3_ internal standard and analyzed via LC-MS/MS. The following seeds were tested:

*Seed tested*

Audubon Park No Waste Blend (Batch2021 I1S)

Country Road Wild Bird Seed (Batch 05620)

Harvest Seed & Supply Orchard Blend (Batch 15317)

Scott Pet Superior Blend Wild Bird Seed (Batch 33619)

Country Pride Wild Bird Food Lot (2021 I5D, Menards *primary bait seed*)

*Captive organic seed tested*

Food to Live Organic Sunflower Seeds

Food to Live Organic Millet

Validating imidacloprid in plasma, dosing solution, and bird seed

The LC-MS/MS was equipped with a Zorbax Eclipse XDB-C18 column (Agilent Technologies; 2.1 mm × 150 mm, 3.5 µm) for separation. Analysis was completed in the positive ion mode following electrospray ionization. The mobile phase consisted of (A) 0.1% formic acid in water and (B) acetonitrile. The gradient began at 98% A and 2% B and was held for 2 min before ramping up to 100% B over 10 min. The mobile phase was kept at 100% B for 2 min before being brought back to initial conditions for re-equilibration (22 min total run time). Further method parameters were as follows: injection volume was 10 µL, column flow rate was 0.4 mL/min, column temperature was 30°C, drying gas temperature was 350°C, gas flow was 10 L/min, nebulizer pressure was 40 psi, and capillary voltage was 4000 V.

**Table S1.** Validation results for the dosing solution and one plasma sample that contained 5-OH-imidacloprid and imidacloprid olefin and recovery of a mass-labeled surrogate (imidacloprid-d4), and the concentrations detected.

| Site | Medium | Extraction Amount (µL or g) | Imidacloprid-d4 (% Recovery) | Imidacloprid (ng/mL or ng/g) | Imidacloprid, 5-OH (ng/mL or ng/g) | Imidacloprid Olefin (ng/mL or ng/g) |
| --- | --- | --- | --- | --- | --- | --- |
| 232170780 | Plasma | 24 | 91.6 | 0 | 46.67 | 73.23 |
